# Knowledge, attitudes, and practices regarding nutritional management in patients with chronic obstructive pulmonary disease: a cross-sectional study in rural China

**DOI:** 10.3389/fnut.2025.1559694

**Published:** 2025-05-16

**Authors:** Xu Zhang, Yueqin Xu, Yongdi Duan, Hua Yi, Wei Luo

**Affiliations:** ^1^Pulmonary and Critical Care Medicine, The First Affiliated Hospital of Yangtze University/The First People's Hospital of Jingzhou, Jingzhou, China; ^2^Integrated Traditional Chinese Medicine and Western Medicine, The First Affiliated Hospital of Yangtze University/The First People's Hospital of Jingzhou, Jingzhou, China; ^3^Pulmonary and Critical Care Medicine, Jianli Hospital of Traditional Chinese Medicine, Jianli, China; ^4^Pulmonary and Critical Care Medicine, The Second People's Hospital of Songzi, Songzi, China; ^5^Central Laboratory, The First Affiliated Hospital of Yangtze University/The First People's Hospital of Jingzhou, Jingzhou, China

**Keywords:** chronic obstructive pulmonary disease, nutritional management, knowledge, attitudes, practice, cross-sectional study, structural equation modeling

## Abstract

**Introduction:**

This study aimed to evaluate the knowledge, attitudes, and practices (KAP) of patients with chronic obstructive pulmonary disease (COPD) concerning nutritional management.

**Methods:**

A cross-sectional study was conducted between June and August 2024, involving COPD patients from six hospitals in Jingzhou. Demographic information and KAP scores were collected and analyzed using a self-developed questionnaire.

**Results:**

A total of 411 valid cases were collected, with a valid response rate of 86.16%. Of the participants, 319 (77.62%) were male, 115 (27.98%) had been diagnosed with COPD for more than 10 years, and 316 (76.89%) had received education on nutritional management. The average scores for knowledge, attitudes, and practices were 7.19±5.13 (range: 0–18), 24.81±4.43 (range: 8–40), and 19.51±5.68 (range: 7–35), respectively. Correlation analysis indicated significant positive relationships between knowledge and attitudes (r = 0.629, *P* < 0.001), knowledge and practices (r = 0.539, *P* < 0.001), and attitudes and practices (r = 0.501, *P* < 0.001). Structural equation modeling revealed that knowledge directly influenced attitudes (β = 0.764, *P* < 0.001) and practices (β = 0.521, *P* < 0.001), while attitudes also directly impacted practices (β = 0.409, *P* < 0.001).

**Conclusion:**

Patients with COPD demonstrated inadequate knowledge, negative attitudes, and inactive practices regarding nutritional management, highlighting a significant gap in effective dietary education. To enhance clinical outcomes, healthcare providers should implement targeted educational programs that emphasize the importance of nutritional management for COPD patients.

## Introduction

Chronic obstructive pulmonary disease (COPD), marked by airflow limitation and persistent respiratory symptoms, affects approximately 10.3% of the global population, with 391 million patients worldwide, of whom more than 80% reside in developing countries (1). In China, the prevalence of COPD is estimated at 13.7%, equating to around 99.9 million patients (2). With rising life expectancy and changes in lifestyle, COPD remains a leading cause of morbidity and mortality, posing serious public health challenges and significant economic and social burdens (3, 4).

Unhealthy dietary patterns, such as the Western diet high in fat, sugar, and processed meats, have been linked to an increased risk of COPD. In contrast, the Mediterranean diet, characterized by a high intake of fruits, vegetables, whole grains, and fish, has been associated with improved lung function, particularly among smokers (5–7). Malnutrition, commonly observed among COPD patients, is characterized by weight loss and muscle wasting and is associated with accelerated disease progression, increased symptom severity, and higher hospitalization rates. Loss of body weight and muscle mass further leads to diminished exercise capacity, reduced quality of life, and increased mortality risk (8). Nutritional interventions, including multi-nutrient and single-nutrient supplements such as protein, vitamin D, and n-3 fatty acids, have demonstrated benefits in improving body weight, muscle mass, and clinical symptoms, reducing inflammation, slowing disease progression, and decreasing the risk of acute exacerbations (9, 10).

The Knowledge, Attitude, and Practices (KAP) survey is a widely used tool to assess awareness, beliefs, and behaviors related to health, based on the premise that increased knowledge positively influences attitudes, which subsequently shape behaviors (11). Given the high prevalence of COPD in China, effective nutritional management tailored to patient needs is essential to improve outcomes. Nutritional deficiencies not only worsen respiratory symptoms but also contribute to poorer overall health outcomes. In China, awareness and management of COPD are evolving, with growing emphasis on incorporating dietary considerations into comprehensive care strategies (12). Effective case finding and management are crucial to ensure that patients receive holistic care, including nutritional counseling, as part of an integrated treatment approach (13–15).

Existing KAP studies on COPD patients primarily focus on disease diagnosis and treatment (16), pulmonary rehabilitation (17), and self-management (18), while limited attention has been given to their knowledge, attitudes, and practices regarding nutrition management. To our knowledge, no KAP study on this topic has been conducted, especially in China. Therefore, this study aims to explore the knowledge, attitudes, and practices of COPD patients concerning nutritional management.

## Materials and methods

### Study design and patients

This cross-sectional study was conducted on patients with COPD from June to August 2024 across six hospitals in Jingzhou, with coordination led by the First Affiliated Hospital of Yangtze University. Ethical approval for the study was obtained from the Medical Ethics Committee of the First Affiliated Hospital of Yangtze University (Approval No.: KY202443), and informed consent was collected from all participants. Inclusion criteria were as follows: (1) a confirmed diagnosis of COPD (19, 20); (2) age of 18 years or older; (3) the ability to comprehend the purpose of the study and voluntarily provide informed consent; and (4) intact consciousness and adequate cognitive function to understand and respond to the questionnaire items. Exclusion criteria included the presence of comorbidities such as advanced cancer or severe cardiac disease to minimize potential confounding effects on nutritional status and maintain cohort homogeneity.

### Questionnaire

The questionnaire design was informed by previous literature (21) and relevant guidelines, including the *Nutritional assessment and therapy in COPD: a European Respiratory Society statement* *(**22**)* and the *Evidence-based practice guidelines for clinical rehabilitation of chronic obstructive pulmonary disease* *(**23**)*. After drafting the initial version, expert feedback was sought, and revisions were made accordingly. The expert panel consisted of three specialists from the Department of Pulmonary and Critical Care Medicine and one clinical nutrition expert, all of whom have over 20 years of professional experience. The experts recommended adding questions regarding smoking status, height, and weight to the demographic section of the questionnaire. Additionally, they revised questions in the survey where there was repetition or unclear language. Through expert consultation, the content validity of the questionnaire was ensured, ensuring that the research design and measurement tools were robust and accurate for the study's objectives. A pilot test with 31 participants was conducted. The Cronbach's α coefficient for the overall questionnaire was 0.945, while the Cronbach's α values for the knowledge, attitude, and practice dimensions were 0.947, 0.857, and 0.945, respectively, demonstrating good reliability across all dimensions.

The final version of the questionnaire, administered in Chinese, consisted of four sections: demographic data (e.g., age, gender, education, occupation, family per capita monthly income, duration of COPD, comorbidities, smoking status, height, weight, Nutritional Risk Screening 2002 score), knowledge, attitudes, and practice dimensions. Body mass index (BMI) was calculated as BMI = weight (kg)/height (m)^2^. The Modified Medical Research Council (mMRC) Dyspnea Scale was used to quantify the severity of breathlessness in patients (24). Nutritional Risk Screening 2002 (NRS-2002) is a tool designed to identify patients at risk for malnutrition (25). It evaluates both nutritional status and disease severity, as these factors significantly influence a patient's nutritional requirements. The screening process includes three components: initial screening questions, an assessment of nutritional status (including weight loss, body mass index, and dietary intake), and an evaluation of disease severity. A cumulative score is calculated, with a score of ≥3 indicating a patient is at risk for malnutrition.

The knowledge dimension contained 9 items, with scores assigned as follows: 2 points for “very familiar,” 1 point for “heard of it,” and 0 points for “unclear,” resulting in a total score range of 0–18. The attitude dimension comprised 8 items using a five-point Likert scale, with scores ranging from 5 (“strongly agree”) to 1 (“strongly disagree”), yielding a total score range of 8-40. The practice dimension included seven items, scored from 1 (“never”) to 5 (“always”), with a total score range of 7–35. The 8th and 9th item in the practice dimension was analyzed descriptively. A threshold of ≥70.0% was used to indicate good knowledge, positive attitudes, and proactive behaviors (26, 27). The questionnaire also included a general knowledge trap question designed to exclude invalid responses from participants who answered without carefully reading the questions.

### Questionnaire distribution and quality control

The questionnaires were distributed online using the Sojump website (https://www.wjx.cn/), where a QR code was generated for easy access. Doctors at each hospital shared the QR code with participants in both outpatient and inpatient settings. Participants scanned the QR code to access and complete the questionnaire. To ensure data quality and completeness, each IP address was restricted to a single submission, and all questions were mandatory. If participants experienced any difficulties while completing the questionnaire, research team members were available to provide explanations and assistance. Questionnaires were deemed invalid and excluded if the completion time was < 90 s, if trap questions were answered incorrectly, or if logical inconsistencies or response patterns indicating repetition were identified.

### Sample size

The sample size was calculated using the formula for cross-sectional studies (28): *n* = $\frac{z^{2}pq}{e^{2}}$, where n represents the number of participants, z is 1.96 for a 95% confidence interval, p is the expected proportion, q is 1-p, and e is the margin of error set at 5%. A conservative estimate of 50% was chosen for p to maximize the sample size. As a result, the calculated sample size for this study was 384 participants.

### Statistical methods

Data analysis was conducted using SPSS 27.0 (IBM, Armonk, NY, USA) and AMOS 26.0 (IBM, Armonk, NY, USA). Normality tests were performed for the distribution of scores in each dimension. For normally distributed data, mean and standard deviation (SD) were used, while median, 25th percentile, and 75th percentile were reported for non-normally distributed data. When comparing two groups, the *t*-test or ANOVA were used for normally distributed continuous variables, while the Wilcoxon-Mann-Whitney test and the Kruskal-Wallis test were applied for non-normally distributed variables. Correlation analysis between dimension scores utilized Spearman correlation coefficients. Structural equation modeling (SEM) was applied within the KAP theoretical framework to assess whether attitudes mediate the relationship between knowledge and practices, calculating the sizes of direct and indirect effects. Model fit was evaluated using the following criteria: Root Mean Square Error of Approximation (RMSEA) < 0.08, Standardized Root Mean Square Residual (SRMR) < 0.08, Tucker-Lewis Index (TLI) > 0.8, and Comparative Fit Index (CFI) > 0.8. A two-tailed *p* < 0.05 was considered statistically significant.

## Results

A total of 477 questionnaires were initially collected. Of these, 62 were excluded due to incorrect responses to trap questions, and 4 were excluded due to abnormally high BMI. Participants with extreme BMI values were excluded as they may indicate data entry errors or underlying medical conditions unrelated to COPD. Finally, a total of 411 valid cases were included, yielding a valid response rate of 86.16%.

### Demographic information and KAP scores of the patients

Among the participants, 319 (77.62%) were male, with a mean age of 66.86 ± 12.89 years. A total of 345 (83.94%) had an education level of middle school or below, 255 (62.04%) had no stable income, 115 (27.98%) had COPD for more than 10 years, 76 (18.49%) were current smokers, and 316 (76.89%) had received education about nutritional management.

The mean scores for knowledge, attitudes, and practices were 7.19 ± 5.13 (range: 0-18), 24.81 ± 4.43 (range: 8-40), and 19.51 ± 5.68 (range: 7-35), respectively. Using a 70% cutoff value, COPD patients were found to show inadequate knowledge, negative attitudes, and inactive practices regarding nutritional management. Differences in participants' knowledge, attitudes, and practice scores were significant across age, family per capita monthly income, and BMI (all P < 0.05). Besides, knowledge scores varied significantly by education level (*P* < 0.001), income type (*P* = 0.002), duration of COPD (*P* = 0.008), comorbidities (*P* = 0.019), severity of symptoms in daily life (*P* < 0.001), and received education on nutritional management (*P* < 0.001). Attitude scores differed based on duration of COPD (*P* = 0.003), smoking status (*P* = 0.046), severity of symptoms in daily life (*P* < 0.001), and received education on nutritional management (*P* < 0.001). Practice scores showed significant variation according to education (*P* < 0.001), marital status (*P* = 0.007), income type (*P* < 0.001), and smoking status (*P* = 0.028) (Table 1).

**Table 1:** Demographic information and KAP scores among the patients.

**Characteristics**	***n* (%)**	**Knowledge**		**Attitude**		**Practice**	
		**Mean** ±**SD**	***P***	**Mean** ±**SD**	***P***	**Mean** ±**-**+**SD**	***P***
**Total**	411	7.19 ± 5.13		24.81 ± 4.43		19.51 ± 5.68	
**Gender**			0.160		0.440		0.533
Male	319 (77.62)	7.39 ± 5.17		24.71 ± 4.52		19.42 ± 5.66	
Female	92 (22.38)	6.53 ± 4.94		25.12 ± 4.10		19.84 ± 5.77	
**Age (years old)**	66.86 ± 12.89		< 0.001		0.034		< 0.001
≤ 50	40 (9.73)	10.85 ± 5.40		26.83 ± 4.47		26.38 ± 6.86	
51–60	57 (13.87)	7.93 ± 5.40		24.51 ± 4.08		20.77 ± 5.53	
61–70	133 (32.36)	6.54 ± 4.86		24.35 ± 4.37		18.95 ± 4.51	
71–80	138 (33.58)	6.07 ± 5.07		24.67 ± 4.58		17.64 ± 5.05	
>80	43 (10.46)	8.40 ± 3.48		25.14 ± 4.13		19.21 ± 4.86	
**Residence**			0.108		0.185		0.353
Rural	394 (95.86)	7.28 ± 5.12		24.87 ± 4.44		19.46 ± 5.71	
urban	17 (4.14)	5.24 ± 5.19		23.41 ± 3.99		20.76 ± 4.82	
**Education level**			< 0.001		0.489		< 0.001
Middle school and below	345 (83.94)	6.81 ± 4.89		24.74 ± 4.35		18.51 ± 5.04	
High school and above	66 (16.06)	9.23 ± 5.88		25.15 ± 4.83		24.76 ± 5.99	
**Marital status**			0.708		0.328		0.007
Married	381 (92.70)	7.17 ± 5.10		24.75 ± 4.46		19.3 ± 5.53	
Other (Single/divorced/widowed)	30 (7.30)	7.53 ± 5.53		25.57 ± 3.99		22.2 ± 6.88	
**Income type**			0.002		0.133		< 0.001
Stable income	156 (37.96)	8.18 ± 5.27		25.22 ± 4.20		21.28 ± 5.82	
Unstable income	255 (62.04)	6.59 ± 4.95		24.55 ± 4.54		18.43 ± 5.32	
**Family's per capita monthly income (Yuan)**			< 0.001		0.045		< 0.001
< 2,000	205 (49.88)	8.06 ± 4.82		25.28 ± 4.71		18.74 ± 5.63	
2,000–5,000	142 (34.55)	5.95 ± 5.12		24.08 ± 3.95		19.50 ± 5.66	
>5,000	64 (15.57)	7.19 ± 5.60		24.88 ± 4.32		22.02 ± 5.22	
**Duration of COPD**			0.008		0.003		0.152
< 6 months	116 (28.22)	8.47 ± 4.96		26.02 ± 4.09		20.27 ± 6.90	
6 months to 3 years	79 (19.22)	6.05 ± 5.33		23.96 ± 3.80		18.39 ± 4.51	
3–10 years	101 (24.57)	6.84 ± 5.72		24.21 ± 5.00		19.35 ± 5.94	
More than 10 years	115 (27.98)	7.00 ± 4.34		24.69 ± 4.41		19.66 ± 4.65	
**Smoking status**			0.863		0.046		0.028
Never	156 (37.96)	7.17 ± 5.72		25.45 ± 4.49		20.14 ± 6.07	
Used to smoke, but quit	179 (43.55)	7.32 ± 4.54		24.58 ± 3.99		18.66 ± 5.18	
Currently smoking	76 (18.49)	6.95 ± 5.20		24.03 ± 5.10		20.22 ± 5.78	
**Comorbidities (hypertension, coronary heart disease, diabetes, cerebrovascular disease)**			0.019		0.369		0.984
Yes	168 (40.88)	7.90 ± 5.13		25.04 ± 4.47		19.52 ± 5.24	
No	243 (59.12)	6.70 ± 5.08		24.64 ± 4.39		19.51 ± 5.97	
Grade 0	148 (36.01)	8.41 ± 4.63		25.68 ± 3.97		19.70 ± 6.52	
Grade 1	131 (31.87)	5.65 ± 4.19		23.55 ± 3.61		18.79 ± 3.93	
Grade 2	91 (22.14)	7.27 ± 6.01		25.23 ± 5.36		20.04 ± 6.16	
Grade 3	34 (8.27)	7.50 ± 6.57		25.35 ± 5.23		20.09 ± 6.10	
Grade 4	7 (1.70)	8.00 ± 4.97		21.71 ± 4.35		19.29 ± 5.99	
**BMI**			< 0.001		< 0.001		0.002
< 18.50	87 (21.17)	8.95 ± 5.34		26.44 ± 4.96		21.23 ± 5.58	
18.5–23.9	251 (61.07)	6.98 ± 4.73		24.59 ± 4.18		18.78 ± 5.57	
≥24.0	73 (17.76)	5.85 ± 5.69		23.62 ± 4.10		19.99 ± 5.74	
**NRS2002**			0.337		0.733		0.115
0–2	379 (92.21)	7.12 ± 5.10		24.78 ± 4.40		19.38 ± 5.65	
≥3	32 (7.79)	8.03 ± 5.50		25.06 ± 4.80		21.03 ± 5.94	
**Received education or information on nutritional management from a hospital or other healthcare institutions**			< 0.001		< 0.001		0.585
Yes	316 (76.89)	8.24 ± 4.90		25.33 ± 4.50		19.59 ± 5.89	
No	95 (23.11)	3.73 ± 4.29		23.05 ± 3.66		19.23 ± 4.96	

### Distribution of responses to knowledge, attitude, and practice

In the knowledge dimension, the three questions most frequently answered as “Unclear” were: “COPD patients should pay special attention to vitamin D supplementation” (K7) with 45.50%, “COPD patients need to increase their protein intake” (K6) with 41.85%, and “COPD patients require more daily energy than the average person” (K5) with 38.20% (Table 2). Regarding attitudes, 12.65% of participants were not worried about potential malnutrition (A1), 12.17% were unwilling to use parenteral nutrition (A6), and 12.17% were not willing to invest time and money in better nutritional management (A8) (Table 3). In terms of practices, 9.49% never used nutritional supplements to improve their nutritional status (P6), 8.52% did not pay attention to vitamin D supplementation (P5), and 8.27% did not maintain regular physical activity (P7) (Table 4). Furthermore, 68.61% of respondents reported limited knowledge about nutritional management, 52.07% cited a lack of time to meet nutritional requirements, and 58.64% perceived the financial burden as too high (P8). When asked about their sources of nutritional management knowledge (P9), 87.35% of participants reported hospital education as a primary source, 58.39% mentioned community outreach programs, 42.58% relied on social media platforms, online health forums, and internet-based educational resources, 49.64% accessed multimedia resources, and 53.53% learned from discussions with relatives, friends, or fellow patients (Supplementary Figure S1).

**Table 2:** Knowledge dimension.

**Items**	**Very familiar, *n* (%)**	**Heard of it, *n* (%)**	**Unclear, *n* (%)**
1. Dietary patterns can influence the progression of COPD. A healthy diet is beneficial for lung function, while an unhealthy diet increases the risk of developing COPD.	72 (17.52)	243 (59.12)	96 (23.36)
2. COPD patients should maintain a healthy and balanced diet. If malnutrition occurs, nutritional supplementation should be considered based on the evaluation of a nutritionist.	77 (18.73)	199 (48.42)	135 (32.85)
3. Malnutrition can lead to weight loss, accelerate muscle and bone tissue consumption, affect the quality of life, and increase the risk of hospitalization.	83 (20.19)	201 (48.91)	127 (30.90)
4. Nutritional management for COPD patients' needs to be individualized. The nutritional management plan differs between malnourished and obese patients.	72 (17.52)	193 (46.96)	146 (35.52)
5. COPD patients require more daily energy than the average person.	55 (13.38)	199 (48.42)	157 (38.20)
6. COPD patients need to increase their protein intake.	54 (13.14)	185 (45.01)	172 (41.85)
7. COPD patients should pay special attention to vitamin D supplementation.	56 (13.63)	168 (40.88)	187 (45.50)
8. COPD patients in good nutritional status and without nutritional risk do not need routine nutritional therapy.	70 (17.03)	185 (45.01)	156 (37.96)
9. For COPD patients who can eat, they should be encouraged to eat on their own. For those who cannot eat orally, parenteral nutrition can be provided.	49 (11.92)	208 (50.61)	154 (37.47)

**Table 3:** Attitude dimension.

**Items**	**Strongly agree, *n* (%)**	**Agree, *n* (%)**	**Neutral, *n* (%)**	**Disagree, *n* (%)**	**Strongly disagree, *n* (%)**
1. I often worry that I may be malnourished.	43 (10.46)	164 (39.90)	142 (34.55)	52 (12.65)	10 (2.43)
2. I believe that additional nutritional supplements are necessary for the average COPD patient.	57 (13.87)	191 (46.47)	145 (35.28)	14 (3.41)	4 (0.97)
3. I am concerned that malnutrition will worsen my condition.	50 (12.17)	182 (44.28)	142 (34.55)	35 (8.52)	2 (0.49)
4. I am willing to undergo regular nutritional assessments.	49 (11.92)	173 (42.09)	161 (39.17)	23 (5.60)	5 (1.22)
5. I am willing to follow the dietary recommendations of my doctor or nutritionist.	48 (11.68)	186 (45.26)	148 (36.01)	23 (5.60)	6 (1.46)
6. If dietary and enteral nutrition alone cannot meet my nutritional needs, I am willing to use parenteral nutrition.	51 (12.41)	148 (36.01)	157 (38.20)	50 (12.17)	5 (1.22)
7. I believe that good nutritional management is beneficial for the treatment of COPD.	50 (12.17)	193 (46.96)	147 (35.77)	18 (4.38)	3 (0.73)
8. I am willing to invest time and money to achieve better nutritional management.	35 (8.52)	151 (36.74)	169 (41.12)	50 (12.17)	6 (1.46)

**Table 4:** Practice dimension.

**Items**	**Always, *n* (%)**	**Often, *n* (%)**	**Sometimes, *n* (%)**	**Rarely, *n* (%)**	**Never, *n* (%)**
1. I adjust my diet according to the recommendations of my doctor or nutritionist.	20 (4.87)	49 (11.92)	168 (40.88)	156 (37.96)	18 (4.38)
2. I regularly measure my weight and pay attention to body changes.	37 (9.00)	54 (13.14)	159 (38.69)	136 (33.09)	25 (6.08)
3. In daily life, I pay attention to increasing my nutritional intake and ensuring enough protein.	28 (6.81)	59 (14.36)	158 (38.44)	152 (36.98)	14 (3.41)
4. I pay attention to a balanced diet and ensure food variety.	34 (8.27)	64 (15.57)	166 (40.39)	128 (31.14)	19 (4.62)
5. I pay attention to supplementing vitamin D.	28 (6.81)	47 (11.44)	135 (32.85)	166 (40.39)	35 (8.52)
6. I try to use nutritional supplements to improve my health condition.	29 (7.06)	43 (10.46)	150 (36.50)	150 (36.50)	39 (9.49)
7. I maintain daily physical activity and ensure an appropriate amount of exercise.	28 (6.81)	60 (14.60)	149 (36.25)	140 (34.06)	34 (8.27)

### Correlations among KAP

Correlation analysis indicated positive relationships between knowledge and attitudes (r = 0.629, P < 0.001), knowledge and practices (r = 0.539, *P* < 0.001), as well as attitudes and practices (r = 0.501, *P* < 0.001) (Supplementary Table S1).

### SEM

**Figure 1:**
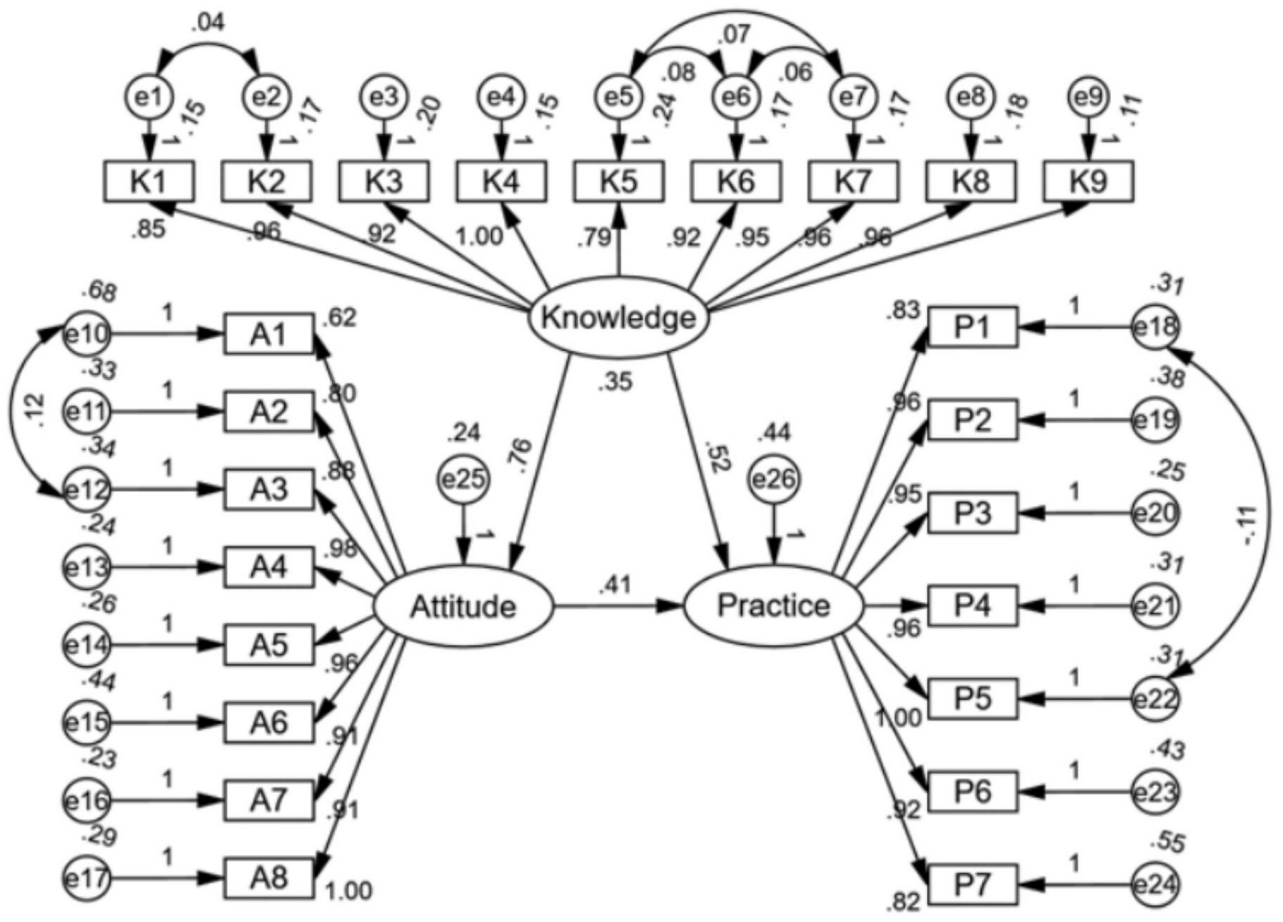
SEM.

The structural model demonstrated a good fit with the following indices: CMIN/DF = 2.765, RMSEA = 0.066, IFI = 0.939, TLI = 0.930, and CFI = 0.939 (Supplementary Table S2). The results of SEM revealed that knowledge directly influenced attitudes (β = 0.764, *P* < 0.001) and practices (β = 0.521, *P* < 0.001), while attitudes also directly impacted practices (β = 0.409, *P* < 0.001) (Supplementary Table S3, Figure 1).

## Discussion

Patients with COPD demonstrated inadequate knowledge, negative attitudes, and inactive practices regarding nutritional management. Strengthening patient education programs focused on improving knowledge and fostering positive attitudes toward nutritional management is recommended to enhance clinical outcomes. Targeted educational interventions should emphasize the role of proper nutrition in disease progression, symptom management, and overall quality of life, ultimately empowering patients to adopt healthier dietary practices and improve long-term prognosis.

The observed inadequacies in knowledge among COPD patients align with findings from other studies, which often report low awareness regarding nutrition's role in managing chronic diseases (29, 30). In particular, many participants in this study were unsure about the increased dietary energy and protein requirements, as well as the importance of vitamin D supplementation, all of which are crucial for managing COPD effectively. This lack of awareness has been similarly documented in other studies, where insufficient nutritional knowledge is linked to lower adherence to dietary recommendations (31, 32). Therefore, enhancing patient education with clear explanations about why protein, energy, and vitamin D are essential for managing COPD is critical. Providing visual aids, easy-to-understand brochures, and video tutorials could make the information more accessible. Monthly nutritional workshops held in collaboration with community health centers can be effective in improving awareness. These workshops might include cooking demonstrations, simple meal planning, and grocery shopping guides specifically tailored for COPD patients (33).

Regarding attitudes, many patients were reluctant to engage in regular nutritional assessments or consider parenteral nutrition, even when dietary and enteral nutrition were insufficient. Similar findings in other chronic disease populations suggest that perceived burden and misconceptions about nutritional interventions contribute to negative attitudes (34, 35). However, evidence shows that patients' attitudes can improve when they receive clear information linking nutrition to improved symptom management and quality of life (36, 37). Personalized counseling sessions that address individual concerns, interactive educational sessions that emphasize the benefits of nutrition in COPD treatment, and the inclusion of patient testimonials may help to correct misconceptions and improve patient engagement.

The inactive practices identified in this study reflect similar patterns reported in other studies, where COPD patients often struggle to adhere to dietary recommendations without consistent professional support (38, 39). Participants in this study rarely adjusted their diet according to medical advice, monitored their weight regularly, or used nutritional supplements. To improve dietary adherence, structured follow-ups with dietitians, the integration of diet plans into electronic medical records for ongoing monitoring, and the provision of at-home nutritional kits or dietary checklists could be beneficial. Offering free or subsidized nutritional counseling and access to affordable nutritional supplements may also help patients overcome financial constraints, which are a known barrier to adherence (40).

Several demographic and clinical variables were significantly associated with differences in KAP scores. Younger patients had better knowledge and practice scores, potentially due to greater access to health information and higher health literacy (41, 42). For older patients, using simplified educational materials, involving caregivers in education, and providing at-home nutritional support could be more effective. Furthermore, disease severity plays a crucial role in shaping patients' attitudes and practices regarding nutritional management. Patients with more severe COPD symptoms often exhibit greater concern about their nutritional status; however, this does not always translate into proactive behaviors (43). Those with more advanced disease may face additional barriers, such as fatigue, dyspnea, and reduced appetite, which hinder adherence to recommended dietary practices. Patients with higher educational levels scored better in knowledge and practice, emphasizing the role of health literacy. Tailoring educational materials to different literacy levels, with more pictorial content for those with lower literacy, could enhance comprehension. Financial stability was also a key factor influencing KAP scores, with patients having a stable income showing better adherence to nutritional practices. This suggests that addressing financial barriers, such as by offering subsidized nutritional supplements and free dietary consultations, is essential for improving nutritional management among patients with unstable income (44, 45).

The positive correlations among knowledge, attitudes, and practices, as indicated by correlation analysis and SEM, align with previous studies showing that improved knowledge serves as the foundation for better attitudes and practices (46, 47). Studies on COPD and other chronic diseases consistently highlight the central role of knowledge in influencing patient behavior, as greater awareness tends to foster a more proactive approach toward disease management (48, 49). In the context of nutritional management, higher knowledge levels have been linked not only to better dietary adherence but also to increased patient confidence in managing symptoms through nutritional interventions (50, 51). Knowledge had the strongest direct influence on attitudes and practices, underscoring the importance of educational interventions. Enhancing patient knowledge through practical, resource-sensitive approaches is likely to yield broader improvements in both attitudes and practices. The SEM results also suggest that knowledge has both direct and indirect effects on practice, mediated by changes in attitudes. This mediating effect is similar to findings reported in studies on diabetes management, where educational interventions significantly improved patients' willingness to follow dietary recommendations by positively altering their perceptions of nutritional benefits (52, 53). By integrating comprehensive educational interventions that are sensitive to the patients' socioeconomic context and health literacy levels, the potential for broader improvements in both attitudes and practices can be realized (54, 55). In particular, community-based interventions that include family members and caregivers may offer additional support, as involvement from social networks has been positively associated with better adherence to dietary regimens. Overall, strengthening the knowledge base among COPD patients through targeted interventions can serve as a critical strategy for improving both attitudes toward and adherence to nutritional management practices. Healthcare providers should integrate discussions on nutrition into routine COPD management, reinforcing its importance alongside pharmacological treatment during each patient visit (56). Similar to medication adherence, regular reinforcement of dietary guidelines may improve long-term nutritional practices and overall disease management.

This study has several limitations. First, its cross-sectional design limits the ability to establish causal relationships between knowledge, attitudes, and practices regarding nutritional management. Second, the use of self-reported questionnaires may introduce response bias, potentially affecting the accuracy of the reported KAP levels. Third, the study was conducted in only six hospitals in Jingzhou, which may limit the generalizability of the findings to other regions or healthcare settings.

## Conclusion

In conclusion, patients with COPD demonstrated inadequate knowledge, negative attitudes, and inactive practices regarding nutritional management, highlighting a gap in their understanding and implementation of dietary strategies. To enhance clinical outcomes, targeted educational interventions focusing on nutritional management should be integrated into routine COPD care to improve patients' knowledge, attitudes.

## Data Availability

The original contributions presented in the study are included in the article/Supplementary material, further inquiries can be directed to the corresponding author.

## Supplementary material

Supplementary Figure S1 Challenges in nutritional management (P8) and sources of knowledge (P9) among COPD patients.

Supplementary Table S1 Correlation analysis of knowledge, attitudes, and practices.

Supplementary Table S2 SEM fit indices.

Supplementary Table S3 SEM.
