# Supplementary figures and images for: Knowledge, attitudes, and practices regarding nutritional management in patients with chronic obstructive pulmonary disease: a cross-sectional study in rural China
Source: Front Nutr. 2025 May 16;12:1559694. doi: 10.3389/fnut.2025.1559694 (PMC12123360; doi:10.3389/fnut.2025.1559694)

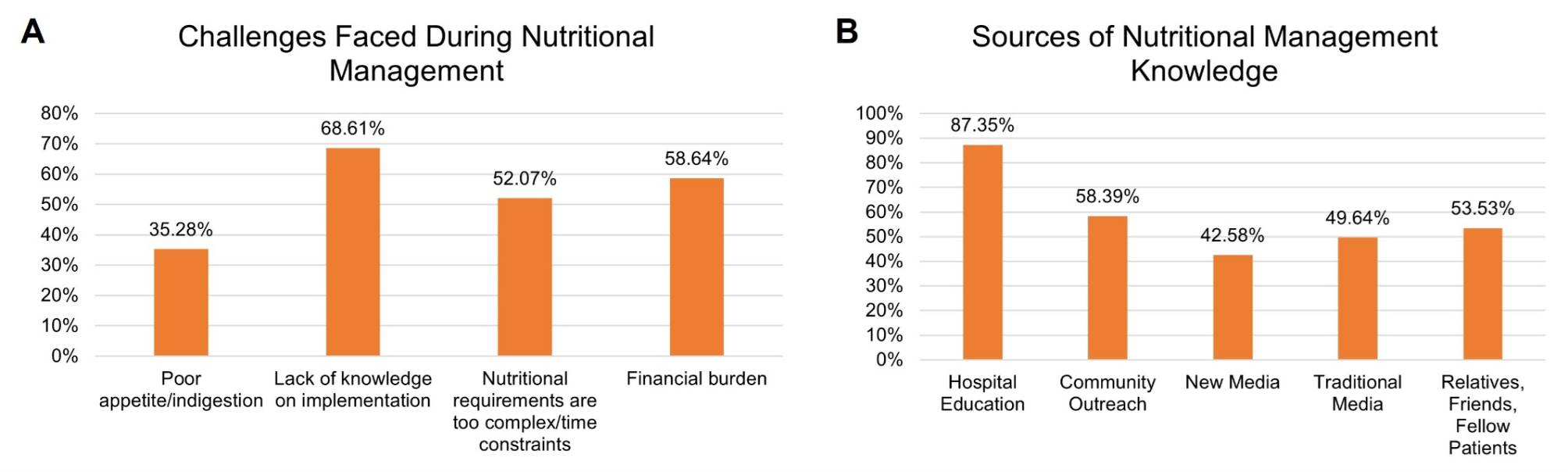

Supplement: Supplementary Figure S1 — Challenges in nutritional management (P8) and sources of knowledge (P9) among COPD patients. [file Image_1.jpeg]
