# Supplementary material for: Knowledge, attitudes, and practices regarding nutritional management in patients with chronic obstructive pulmonary disease: a cross-sectional study in rural China
Source: Front Nutr. 2025 May 16;12:1559694. doi: 10.3389/fnut.2025.1559694 (PMC12123360; doi:10.3389/fnut.2025.1559694)
Supplement: Supplementary Table S1 — Correlation analysis of knowledge, attitudes, and practices. [file Table_1.docx]

Table S1 Correlation Analysis of Knowledge, Attitudes, and Practices

|  | **Knowledge** | **Attitude** | **Practice** |
| --- | --- | --- | --- |
| **Knowledge** | 1 |  |  |
| **Attitude** | 0.629 (P<0.001) | 1 |  |
| **Practice** | 0.539 (P<0.001) | 0.501 (P<0.001) | 1 |

Table S2. SEM fit indices

| **Indicators** | **Reference** | **Actual** |
| --- | --- | --- |
| CMIN/DF | 1-3: Excellent, 3-5: Good | 2.765 |
| RMSEA | <0.08: Good | 0.066 |
| IFI | >0.8: Good | 0.939 |
| TLI | >0.8: Good | 0.930 |
| CFI | >0.8: Good | 0.939 |

Table S3. SEM

|  |  |  | **β** | **P** |
| --- | --- | --- | --- | --- |
| Attitude | <--- | Knowledge | 0.764 | <0.001 |
| Practice | <--- | Attitude | 0.409 | <0.001 |
| Practice | <--- | Knowledge | 0.521 | <0.001 |
| K1 | <--- | Knowledge | 0.850 | <0.001 |
| K2 | <--- | Knowledge | 0.961 | <0.001 |
| K3 | <--- | Knowledge | 0.924 | <0.001 |
| K4 | <--- | Knowledge | 1.000 |  |
| K5 | <--- | Knowledge | 0.790 | <0.001 |
| K6 | <--- | Knowledge | 0.923 | <0.001 |
| K7 | <--- | Knowledge | 0.950 | <0.001 |
| K8 | <--- | Knowledge | 0.963 | <0.001 |
| K9 | <--- | Knowledge | 0.962 | <0.001 |
| A8 | <--- | Attitude | 1.000 |  |
| A7 | <--- | Attitude | 0.912 | <0.001 |
| A6 | <--- | Attitude | 0.911 | <0.001 |
| A5 | <--- | Attitude | 0.956 | <0.001 |
| A4 | <--- | Attitude | 0.976 | <0.001 |
| A3 | <--- | Attitude | 0.880 | <0.001 |
| A2 | <--- | Attitude | 0.798 | <0.001 |
| A1 | <--- | Attitude | 0.621 | <0.001 |
| P1 | <--- | Practice | 0.830 | <0.001 |
| P2 | <--- | Practice | 0.962 | <0.001 |
| P3 | <--- | Practice | 0.950 | <0.001 |
| P4 | <--- | Practice | 0.961 | <0.001 |
| P5 | <--- | Practice | 1.000 |  |
| P6 | <--- | Practice | 0.916 | <0.001 |
| P7 | <--- | Practice | 0.824 | <0.001 |
